# Self-reported sexual orientation among undergraduates of 10 universities in Guangzhou, China

**DOI:** 10.1371/journal.pone.0201817

**Published:** 2018-08-24

**Authors:** Yuan Yan, Shuiyuan Xiao, Haihong Liu, Pierre Chue

**Affiliations:** 1 Mental Health Center, Xiangya Hospital, Central South University, Changsha, Hunan Province, China; 2 Psycho-education and Counseling Center, Guangdong University of Technology, Guangzhou, Guangdong Province, China; 3 Department of Social Medicine and Health Management, Xiangya School of Public Health, Central South University, Changsha, Hunan Province, China; 4 Department of Psychiatry, University of Alberta, Edmonton, Alberta, Canada; The University of Warwick, UNITED KINGDOM

## Abstract

Few studies have investigated the distribution of sexual orientation among Chinese university students and identified the socio-demographic factors associated with sexual orientation. For the present study, we administered a paper-based, 5-point, self-report, sexual orientation scale to a stratified, random sample of 9071 undergraduates across all 10 universities in Guangzhou Higher Education Mega Center, Guangzhou, China. Multivariable ordinal regression analysis was used to explore the relationship between demographic factors and sexual orientation. A total of 8320 respondents completed the survey. Of 8182 valid respondents, 80.6% self-reported as exclusively heterosexual, 12.6% self-reported as mostly heterosexual, 5.4% self-reported as bisexual, 0.7% self-reported as mostly homosexual, and 0.8% self-reported as exclusively homosexual. About one fifth of male students and one fourth of female students reported some degree of divergence from exclusive heterosexuality. This indicates that in China there are a large number of university students who are potentially involved in same-sex sexual attraction.

## Introduction

Sexual orientation largely emerges in adolescence and stabilizes in early adulthood [1] and represents a subjective internal experience which plays a crucial role in the construction of an individual’s self-concept [2]. Sexual orientation, together with culture, plays a role in moderating sex differences in personality traits and occupational preferences [3–5]. Burgeoning research indicates that, compared with exclusively heterosexual undergraduates, students who report other sexual orientation are at higher risk of mental disorders and unhealthy behaviors and experience discrimination, victimization, stigmatization and prejudice [6]. The sexual orientation of youth has attracted interest in many countries, such as the United States of America (USA) and China. In the USA, sexual orientation questions have been added to statewide public health surveillance [7]. In China, sexual orientation of the youth has been described in mass media and in fictional literature but has been largely ignored by the scientific literature. The only study we found was published by Guo et al [8], which evaluated a sample of 22,288 youth aged from 15 to 24 years. The participants were rural and urban residents including university students [8]. In the study by Guo et al., sexual orientation was defined as sexual identity and assessed by three discrete categories: heterosexual, bisexual, and homosexual [8]. The present study is the first to identify the sexual orientation of China’s 37 million undergraduates and is the first to use a measure that reflects the perspective that sexuality lies on a continuum [2, 9].

This paper presented reports on sexual orientation in a large sample of undergraduates across all 10 universities in the Guangzhou Higher Education Mega Center (HEMC). Demographic characteristics are purported to be associated with sexual orientation attitudes [10], so the socio-demographic factors and their association with sexual orientation were also analyzed.

## Methods

A stratified random sampling design was used in this study. Firstly, we obtained the number of undergraduates of each university and the percentage of 121,181 undergraduates of the all 10 universities. Secondly, the target sample size was set as 10,000, and the percentages were used to calculate each university's sample size. Thirdly, randomized sampling was performed at class-level (about 30 students in a class) for each university. Fourthly, the final sample of 9071 university students was obtained. A self-administered questionnaire was given to 9,071 (7.5%) of 121,181 undergraduates across all 10 universities in HEMC in the fall semester of 2015 in Guangzhou, China. The 10 universities are Sun Yat-sen University, South China University of Technology, South China Normal University, Guangzhou University, Guangdong University of Foreign Studies, Guangzhou University of Chinese Medicine, Guangdong Pharmaceutical University, Guangdong University of Technology, Guangzhou Academy of Fine Arts and Xinghai Conservatory of Music. Students of these 10 universities are from all over the country. A total of 8,320 (91.7% of 9,071) students filled in the questionnaires and 8,182 of them responded to the question on sexual orientation, yielding a response rate of 90.2% and a refusal rate of 9.8%. The laboratory protocol was deposited in protocols.io with a digital object identifier link (http://dx.doi.org/10.17504/protocols.io.pcrdiv6).

Informed consent was given verbally by participants at the beginning of the survey outside of school hours. The consent document was read out and any questions on the survey's purpose, content, confidentiality, participant's rights, benefit and risk were answered immediately and discussed. There were no incentives to join the survey and participants were able quit at any time without any risk. Participation was completely voluntary and all personal information was kept strictly confidential for the purpose of the study. The consent procedure and the study were approved by the Ethics Committee, Xiangya School of Public Health, Central South University.

Self-reported sexual orientation was measured using a single item on a 5-point scale with the instruction, "Please circle a number on the line scale which best describes your sexual orientation, 1 indicates exclusively heterosexual, 5 indicates exclusively homosexual” [S1 Supporting Information].

Statistical analysis was firstly used to describe the demographic characteristics of the total sample by self-reported sexual orientation among undergraduate students in Guangzhou, China. Secondly, Chi-square tests were used to determine the association between the demographics and sexual orientation. Thirdly, after the assumption of proportional odds was tested by using Test of Parallel Lines (p>0.05), multivariable ordinal regression analysis was used for examining the association between demographic variables and self-reported sexual orientation. The results are reported as odds ratios (OR) with 95% confidence interval (CI).

## Results

Of the 8,182 valid questionnaires, 3,894 (47.6%) were male, 4,147 (51.0%) were female, and 141 (1.4%) did not report their gender. The average age of participants was 19.8 (SD = 1.38) years, with a range of 15 to 29 years. The proportions of freshman, sophomore, junior and senior was 32.4%, 30.1%, 25.8% and 11.7%, respectively. 52.8% of the sample were majoring in Humanities and Social Sciences, 47.2% were majoring in Natural Sciences (Table 1).

**Table 1:** Demographics of the total sample by self-reported sexual orientation among undergraduate students in Guangzhou, China.

Variables	Subgroups size (%)	Self-reported sexual orientation in subgroups (%)					χ^2^*	df	p
		Exclusively Homosexual	Mostly Homosexual	Bisexual	Mostly Heterosexual	Exclusively Heterosexual			
10 universities sample	8182	64 (0.8)	61 (0.7)	438 (5.4)	1027 (12.6)	6592 (80.6)			
Gender									
Male	3894 (47.6)	39 (1.0)	26 (0.7)	112 (2.9)	261 (6.7)	3456 (88.8)	352.662	4	<0.001
Female	4174 (51.0)	23 (0.6)	35 (0.8)	319 (7.6)	744 (17.8)	3053 (73.1)			
Gender unreported	114 (1.4)	2 (1.8)	0 (0.0)	7 (6.1)	22 (19.3)	83 (72.8)			
Ethnicity									
Han	8030 (98.6)	62 (0.8)	59 (0.7)	413 (5.1)	985 (12.3)	6389 (79.6)	2.604	4	0.626
Other	113 (1.4)	1 (0.9)	1 (0.9)	8 (0.1)	18 (15.9)	83 (73.5)			
Age group									
≤18 years	1440 (17.8)	9(0.6)	16 (1.1)	92 (6.4)	197 (13.7)	1110 (77.1)	31.322	12	0.002
19 years	2055 (25.4)	13 (0.6)	14 (0.7)	117 (5.7)	282 (13.7)	1607 (78.2)			
20 years	2147 (26.5)	20 (1.0)	13 (0.6)	113 (5.3)	265 (12.3)	1699 (79.1)			
≥21 years	2459 (30.4)	20 (0.8)	15 (0.6)	104 (4.2)	256 (10.4)	2023 (82.3)			
Grade level									
Freshman	2681 (32.2)	18 (0.7)	23 (0.9)	126 (4.7)	333 (12.4)	2150 (80.2)	19.158	12	0.085
Sophomore	2512 (30.2)	18 (0.7)	16 (0.6)	144 (5.7)	329 (13.1)	1958 (78.0)			
Junior	2144 (25.8)	16 (0.7)	16 (0.7)	105 (4.9)	273 (12.7)	1699 (79.2)			
Senior	983 (11.8)	12 (1.2)	6 (0.6)	63 (6.4)	92 (9.4)	785 (79.9)			
Major									
Humanities & Social Sciences	4408 (53.0)	37 (0.8)	35 (0.8)	301 (6.8)	643 (14.6)	3308 (75.0)	103.497	4	<0.001
Natural Sciences	3912 (47.0)	27 (0.7)	26 (0.7)	137 (3.5)	384 (9.8)	3284 (83.9)			
Being an only child									
No	2340 (30.2)	20 (0.9)	24 (1.0)	166 (7.1)	339 (14.5)	1753 (74.9)	53.873	4	<0.001
Yes	5406 (69.8)	37 (0.7)	32 (0.6)	235 (4.3)	604 (11.2)	4435 (82.0)			
Original family location									
Countryside	2317 (29.3)	22 (0.9)	11 (0.5)	80 (3.5)	220 (9.5)	1961 (84.6)	95.835	12	<0.001
Town or county	1947 (24.7)	10 (0.5)	10 (0.5)	86 (4.4)	231 (11.9)	1583 (81.3)			
Small-medium city	2371 (30.0)	12 (0.5)	26 (1.1)	148 (6.2)	336 (14.2)	1814 (76.5)			
Metropolis	1263 (16.0)	12 (1.0)	12 (1.0)	100 (7.9)	185 (14.6)	932 (73.8)			
Living with parents prior to university									
Yes	4150 (60.1)	28 (0.7)	24 (0.6)	192 (4.6)	512 (12.3)	3332 (80.3)	54.792	8	<0.001
No	2754 (39.9)	22 (0.8)	21 (0.8)	168 (6.1)	334 (12.1)	2183 (79.3)			
Father’s education									
Primary school or lower	1132 (15.0)	14 (1.2)	9 (0.8)	42 (3.7)	127 (11.2)	940 (83.0)	47.273	8	<0.001
High school	4718 (62.3)	29 (0.6)	26 (0.6)	214 (4.5)	580 (12.3)	3869 (82.0)			
University or college	1719 (22.7)	10 (0.6)	20 (1.2)	138 (8.0)	230 (13.3)	1321 (76.8)			
Mother’s education									
Primary school or lower	2179 (28.8)	22 (1.0)	12 (0.6)	74 (3.4)	239 (11.0)	1832 (84.1)	8.325	4	0.08
High school	4243 (56.0)	21 (0.5)	29 (0.7)	235 (5.5)	543 (12.8)	3415 (80.5)			
University or college	1156 (15.3)	7 (0.6)	11 (1.0)	92 (8.0)	150 (13.0)	896 (77.5)			

* Chi-square tests on sexual orientation in the groups based on variables.

A total of 80.6% respondents self-reported as exclusively heterosexual, 12.6% self-reported as mostly heterosexual, 5.4% self-reported as bisexual, 0.7% self-reported as mostly homosexual, and 0.8% self-reported as exclusively homosexual. The reported sexual orientation of males and females was significantly different. Compared with females, males reported higher rate of exclusively heterosexual (88.8% vs. 73.1%) and exclusively homosexual (1.0% vs. 0.6%) orientation. Students who did not report their gender reported the lowest rate of exclusively heterosexual (72.8%) and highest rate of exclusively homosexual (1.8%) orientation (Table 1).

Multivariable ordinal regression revealed that the students without siblings (OR = 1.44, 95% CI, 1.09–1.92 for male; OR = 1.36, 95% CI, 1.14–1.62 for female) or who were not living with parents prior to university (OR = 0.79, 95% CI for male, 0.63–0.99; OR = 0.86, 95% CI, 0.75–0.99 for female) showed a positive association with same-sex sexual attraction. Female students of lower age groups and from a metropolis, and male students majoring Humanities and Social Sciences (OR = 1.50, 95% CI, 1.19–1.90) were positively associated with same-sex sexual attraction (Table 2).

**Table 2:** Multivariable ordinal regression analysis examining the association between socio-demographic variables and sexual orientation among undergraduate students in Guangzhou, China.

	Female students			Male students		
	OR	95% CI	p	OR	95% CI	p
Age groups (years)						
≤18	1.31	1.06–1.62	0.012	0.90	0.64–1.26	0.522
19	1.29	1.06–1.58	0.010	0.82	0.60–1.11	0.193
20	1.24	0.98–1.52	0.033	0.89	0.66–1.20	0.433
≥21	1			1		
Major						
Humanities & Social Sciences	0.89	0.69–0.76	0.134	1.50	1.19–1.90	0.001
Natural Sciences	1			1		
Being an only child						
No	1.36	1.14–1.62	<0.001	1.44	1010–1.92	0.01
Yes	1			1		
Original family location						
Countryside	0.68	0.53–0.87	0.002	0.85	0.58–1.26	0.426
Town or county	0.80	0.64–0.99	0.043	0.82	0.55–1.22	0.331
Small-medium city	0.88	0.73–1.07	0.202	0.98	0.68–1.40	0.893
Metropolis	1			1		
Living with parents prior to university						
Yes	0.86	0.75–0.99	0.04	0.79	0.62–0.99	0.04
No	1			1		

OR: Odds ratio; 95% CI: 95% confidence interval.

## Discussion

This study indicates that self-reported sexual orientation of university students in China is distributed along a continuum. About one fifth of male students and one fourth of female students self-reported some degree of divergence from exclusive heterosexuality. The proportion of students self-reported as non-exclusively heterosexual was 5 times higher than that of the 2013 US National Health Interview Survey, which involved a sample of 34,557 adults aged 18 and over [11]. Among the non-exclusively heterosexual, the prevalence of exclusively homosexual orientation was similar to a national survey in China for a target age group of 15–24, which was 0.79% [8], but much lower than that of a similar US sample, which was 3.7%; the average age of this sample was 19.8 years [12]. The results imply that in China there are a large number of university students who self-report as non-exclusively heterosexual, which may influence their attitude on same-sex sexual behaviors.

In recent years, same-sex related sexual activities have been increasingly reported as a risk factor of HIV infection in China. A report released by the National Health and Family Planning Commission of China indicates that among young students who are infected with HIV, the proportion attributed to men who have sex with men (MSM) had increased from 58.5% in 2008 to 81.6% in 2014 [13]. Furthermore, in a study of a sample of 1824, 33.8% of male university students who reported having had sex with a man at least once, also had female partners [14]. In these studies, the men who have sex with men and women (MSMW) might be subsumed under the MSM category, and they had distinct sexual risk behaviors from men who have sex with men only (MSMO) [15, 16]. In contrast to Friedman et al’s finding of little evidence to support substantial viral bridging behavior [15], data from National HIV Behavioral Surveillance and 20 US cities showed MSMW may play a potential role in bridging the transmission of HIV to heterosexual women [17–19].

Homosexual orientation, either exclusively or not exclusively, may result in high psychosocial stress to students. There are a few studies that have reported excessive suicidal behavior, depressive and anxiety disorders, and substance use among homosexual students, which may be the result of socio-cultural pressures and intrapersonal conflicts related to their sexual orientation [20–22]. When homosexual students choose to disclose their sexual identity, they have to cope with the socio-cultural stress of homophobic prejudice, social rejection, discrimination, and harassment [23]. Conversely, when they choose not to reveal their sexual identity, their relationships may be based on fear of discovery and duplicity, which can cause insecurity, social withdrawal, and demoralization [23].

This study indicates that being an only child, not living with parents prior to going to university, being female, being younger, being from a metropolis, and majoring in Humanities and Social Sciences (if male) are factors positively related to non-exclusive heterosexuality, i.e. somewhat attracted to the same sex. Previous studies showed that acceptable and/or normal view of homosexuality was associated with living in urban area, higher economic status, higher education level, student status, greater sexual and reproductive health knowledge, preference for Western movies/videos, open attitudes regarding family values, gender roles and premarital sex, and having had same-sex sexual contact [10, 24–26]. It suggests that young students with one or more of these characteristics may have greater likelihood of regarding homosexuality as normal and/or acceptable, and this may influence their attitude towards and disclosure of sexual orientation. However, it is still unclear how sexual orientation, particularly homosexual orientation develops among adolescents and young adults not only in China, but in other countries.

## Limitations

Participants of this study was recruited from the 10 universities of HEMC of Guangzhou. Although the students come from all over the country, there is limited generalizability to Chinese university students as a whole. In addition, there was a refusal rate of 9.8% to the question of sexual orientation, and the lowest rate of exclusively heterosexual (72.8%) and highest rate of exclusively homosexual (1.8%) orientation in the students who did not report their gender. It may suggest a bias on self-reporting, and sexual minorities may be more likely to omit/avoid responses to the question of sexual orientation.

## Conclusions

Our study indicated that of 8182 valid respondents, 80.6% self-reported as exclusively heterosexual, 12.6% self-reported as mostly heterosexual, 5.4% self-reported as bisexual, 0.7% self-reported as mostly homosexual, and 0.8% self-reported as exclusive homosexual. About one fifth of male students and one fourth of female students reported some degree of divergence from exclusive heterosexuality. This indicates that in China there are a large number of university students who are potentially involved in same-sex sexual attraction.

## Supporting information

S1 Supporting Information The survey questions used in the study. (DOCX)
