# Supplementary material for: Self-reported sexual orientation among undergraduates of 10 universities in Guangzhou, China
Source: PLoS One. 2018 Aug 24;13(8):e0201817. doi: 10.1371/journal.pone.0201817 (PMC6108474; doi:10.1371/journal.pone.0201817)
Supplement: S1 Supporting Information — (DOCX) [file pone.0201817.s001.docx]

the survey questions used in the study

(in Chinese)

| 学号 |  |
| --- | --- |
| 姓名（拼音） |  |
| 性别 |  |
| 年龄 |  |
| 民族 |  |
| 电话 |  |
| 邮箱 |  |
| 年级 |  |
| 专业 |  |
| 兄弟姐妹数 |  |
| 家中排行 |  |
| 家庭所在地 | 1=农村；2=乡镇；3=县城；4=小城市；5=中等城市；6=大城市 |
| 父亲教育水平 | 1=未上学；2=小学；3=初中；4=高中；5=大学及以上 |
| 母亲教育水平 | 1=未上学；2=小学；3=初中；4=高中；5=大学及以上 |
| 上大学前是否与父母同住 | 1=是；2=否 |
| 您认为您的性倾向是(请在下面的标尺上圈出一个数字 1=完全异性恋，5=完全同性恋)   \|  \|  \|  \|  \|  \|  \| \| --- \| --- \| --- \| --- \| --- \| --- \| \| 完全异性恋 \| 1 \| 2 \| 3 \| 4 \| 5完全同性恋 \| | |

the survey questions used in the study

(In English)

study ID.

name (spelling in Chinese Pinyin)

sex

age

ethnicity

cell phone number

e-mail address

grade level

major (discipline classification)

number of siblings

your bird order

original family location:

1=countryside 2=town 3=county 4=small city 5=medium city 6=metropolis

father's education level:

1=has not been to school; 2=primary school; 3=junior high school; 4=high school; 5=university or college

mother's education level:

1=has not been to school; 2=primary school; 3=junior high school; 4=high school; 5=university or college

living with parents prior to university

1=yes; 2=no

Please circle a number on the line scale which best describes your sexual orientation, 1 indicates exclusive heterosexual, 5 indicates exclusive homosexual.

|  |  |  |  |  |  |
| --- | --- | --- | --- | --- | --- |
| exclusive heterosexual | 1 | 2 | 3 | 4 | 5 exclusive homosexual |
